# Impact of leaks and ventilation parameters on the efficacy of humidifiers during home ventilation for tracheostomized patients: a bench study

**DOI:** 10.1186/s12890-019-0812-z

**Published:** 2019-02-18

**Authors:** Noémie Haziot, Mohamed Ibrahim, Kaixian Zhu, Charles-Philippe Thevenin, Sebastien Hardy, Jésus Gonzalez-Bermejo

**Affiliations:** 1 Service de Pneumologie et Réanimation Médicale (Département “R3S”)AP-HP, Groupe Hospitalier Pitié-Salpêtrière Charles Foix, 47-83 boulevard de l’hôpital, F-75013 Paris, France; 2 Air Liquide Healthcare (EXPLOR Center), Gentilly, France; 3 Sorbonne Université, UPMC Univ Paris 06, INSERM, UMRS1158 Neurophysiologie respiratoire expérimentale et clinique, Paris, France

**Keywords:** Invasive ventilation, Humidification, Heated humidifier, Vented circuit, Unintentional leak, Tracheostomy, Bench test, Respiratory therapy, Respiration, Artificial

## Abstract

**Background:**

During invasive ventilation, the upper airway is bypassed and no longer participates in humidification of inspired gases, which is essential to avoid harmful consequences such as endotracheal tube occlusion. In the case of increased air flow, especially in the presence of leaks (intentional or unintentional), the humidification provided by humidifiers may become ineffective. The objective of this bench study was to evaluate the quality of humidification provided by heated humidifiers under various home ventilation conditions.

**Methods:**

Five heated humidifiers were tested in eight configurations combining circuit (expiratory valve or vented circuit), tidal volume (600 or 1000 mL) and presence of unintentional leak. Absolute humidity (AH) was measured at the upstream of the test lungs, which were placed in a 34 °C environmental chamber in order to simulate body temperature.

**Results:**

The AH measured in the valve circuit ranged between 30 mg/L and 40 mg/L and three out of the five humidifiers achieved an AH higher than the recommended level (33 mg/L). With the vented circuit without unintentional leak, when tidal volume was set at 600 mL, all humidifiers reached an AH higher than 33 mg/L except one device; when the tidal volume was set at 1000 mL and unintentional leak was present, four out of the five humidifiers provided an AH lower than 33 mg/L.

**Conclusion:**

This study shows that, except under certain home ventilation conditions, such as high tidal volumes with unintentional leak in vented circuit, most heated humidifiers ensure sufficient humidification to avoid the risk of side effect in patients.

**Electronic supplementary material:**

The online version of this article (10.1186/s12890-019-0812-z) contains supplementary material, which is available to authorized users.

## Background

Humidification is essential during invasive ventilation as the upper airway is bypassed and can no longer participates in the necessary humidification of inspired gases [1–3]. Physiologically, the temperature in the carina is about 34 °C with an absolute humidity of about 35 mg/L. In the alveoli, inspired gases reach a temperature of 37 °C with an absolute humidity of 44 mg/L [4–7]. Insufficient humidification can induce many complications, more or less severe and usually reversible [4, 8–13], but some complications, such as endotracheal tube occlusion, can be fatal [14–17]. Sub-occlusions are more frequent and less severe, but they increase the respiratory work, thereby prolonging ventilator weaning. Various humidification systems have therefore been developed, including heated humidifiers and heat and moisture exchangers.

When a heated humidifier is used, it is recommended to achieve an absolute humidity of 33 to 44 mg/L with a temperature between 34 °C and a maximum of 41 °C [18]. In 2017, the International Organization for Standardization published standard ISO 80601-2-74 [19] defining the particular requirements for respiratory humidifying equipment for humidifier manufacturers, i.e. an absolute humidity greater than 33 mg/L.

The efficacy of humidification with a heated humidifier depends on several parameters: the air-water contact surface area, the temperature of the heated water in the reservoir, the air-water contact time and therefore the flow rate in the reservoir. The quality of humidification can therefore be altered by various external factors, such as ventilatory parameters, especially increased inspiratory air flow [4, 20–24], high ambient air temperature [20, 21, 25, 26], the type of ventilator (intensive care ventilators use dry medical gases), the type of ventilatory circuit (increased air flow rate when a vented circuit is used), unintentional leaks, and phonation (leak ventilation). All of these factors may be combined, inducing a considerable increase in air flow as a result of technological progress in ventilator turbines, while maintaining good ventilation, but may alter the quality of humidification.

The objective of this study was to evaluate the quality and efficacy of gas humidification in various invasive ventilation situations during home prolonged ventilation for tracheostomized patients. After having designed an original test bench, the quality of humidification provided by various heated humidifiers was determined in various increased air flow situations.

## Materials and methods

The test bench was designed from January 2014 onwards and the final measurements were performed from March 2016 to April 2017.

Five different home heated humidifiers were tested: MR810, HC550 and HC150 (Fisher & Paykel, Auckland, New Zealand), AIRcon (WILAMED, Kammerstein, Germany) and D900 Humicare (Resmed, Gründler, Freudenstadt, Germany). They were adjusted to the highest level of humidification. They all are indicated in invasive ventilation except for HC150 but which is often incorrectly used in this indication in France.

**Fig. 1:**
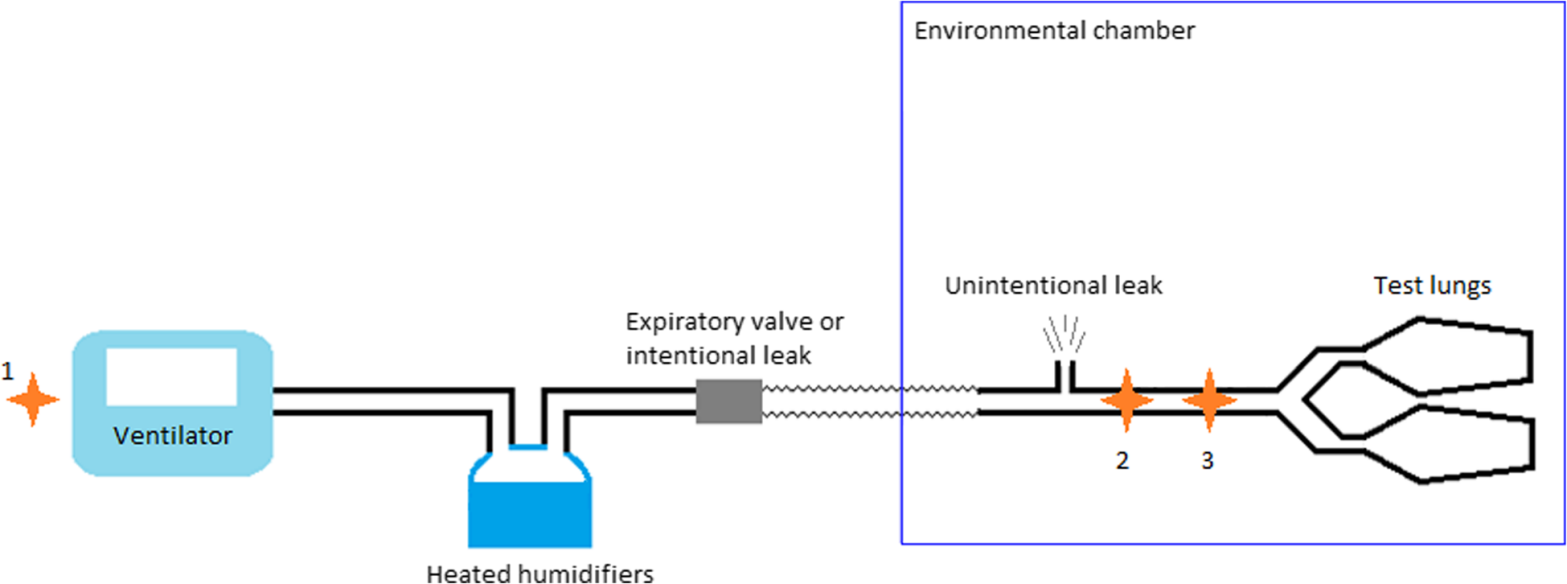
Complete bench test set-up. Schematic representation of the bench with two test lungs placed in a environmental chamber and the ventilatory circuit consisting of the ventilator, the heated humidifiers and, depending of the chosen configuration, an expiratory valve (valve circuit) or an intentional leak (vented circuit). An unintentionale leak can also be created before test lungs. Hygrometric measurement sites: 1 = Hygrometer measuring ambient conditions around the ventilator; 2 = Hygrometer with high humidity level probe (heated probe); 3 = Second hygrometer

The experimental set-up is presented in Fig. 1. The lungs were simulated by two Servo Lung 190 test lungs, each with a capacity of 1 L (Siemens Maquet, Rastatt, Germany). The test lungs were connected by means of a Y-piece, connected to the humidifier via a corrugated tube and the breathing circuit. The test lung set-up was then placed in a CL5–45 environmental chamber (BIA Climatic, Saint Conflans Honorine, France). The temperature of the environmental chamber was adjusted to 34 °C, which corresponds to the temperature at the carina. A Testo 635 hygrometer (Testo, Lenzkirch, Germany) was used to measure temperature and humidity of the air entering the lungs, by placing the tip of the probe (reference 06362135) between the test lungs and the unintentional leak orifice. In the final version of the test bench, to avoid measurement errors due to saturation of this probe, a second hygrometer with a heated probe (high humidity level probe (reference 06362142) and hygrometer Testo 645 Heater Sensor (Testo, Lenzkirch, Germany)) was added at the Y-piece. Circuits were also changed and dried for 15 min after each configuration. These two hygrometers have a response time of 30 s.

Hygrometers were connected by a USB port to the computer and data were acquired by Testo Comfort Software X35 (Testo, Lenzkirch, Germany).

Unintentional leaks were simulated by a 4 mm diameter calibrated orifice resulting in a leak of 24 L/min at 10 cmH2O as found in other studies [27]. The orifice was placed between the test lungs and the corrugated tube. Two types of ventilatory circuits were used: by using an expiratory valve for the valve circuit, or by using a specific connector to create an intentional leak for the vented circuit (Whisper Swivel 2, Respironics, Murrysville, Pennsylvania, USA). A TRILOGY 100 ventilator (Respironics, Murrysville, Pennsylvania, USA) was used in volume-assist control (VAC) mode with a respiratory rate of 12 cycles per minute, positive expiratory pressure (PEEP) of 5 cmH_2_O, an I/E ratio of 1:2, i.e. inspiratory time of 1.6 s, and a square flow pattern.

Eight different configurations were created by combinations of:

- Two levels of tidal volume: 600 mL (standard situation) or 1000 mL (extreme situation as for phonation in tracheostomized patient).
- Two types of breathing circuits: expiratory valve circuit or vented circuit
- Presence or absence of an unintentional leak

Those eight configurations were applied to test the five heated humidifiers, resulting in 40 configurations.

The water consumption of heated humidifiers was determined by weighing the water reservoir before and after each test using a Sartorius precision balance (Type 1564 001, AG Göttingen, Germany). The water consumption was presented as mL/hour. The size of the water reservoir of each heated humidifier being different (400 ml for the MR810, HC550 and HC150; 500 ml for the D900 and 200 ml for the AIRcon), water consumption results are also expressed as the time to empty the water reservoir.

Ambient conditions were monitored during the tests. Relative humidity was monitored with a Logger GL240 hygrometer (Graphtec, Tokyo, Japan) and temperature was monitored with an HI 93532 thermometer (Hanna Instruments, Tanneries, France) with the probes placed in the ventilator air inlet (Fig. 1). Absolute humidity was calculated from the relative humidity and temperature measured by the hygrometers.

**Fig. 2:**
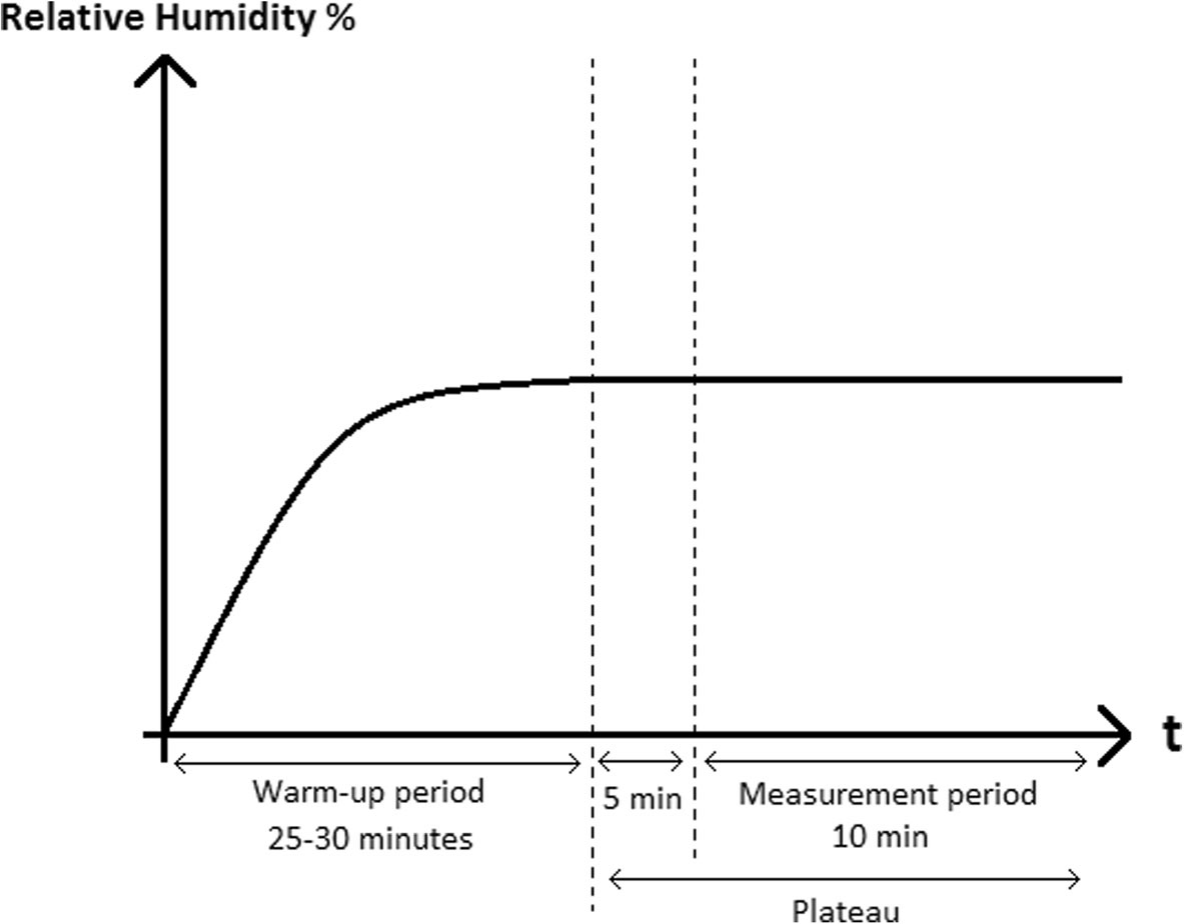
Graph showing the measurement protocol. After a warm-up period of aroud 25–30 min, the relative humidity reaches a plateau (no temperature variation greater than 1 °C and relative humidity variation greater than 5%). The measurement period begins 5 min after the plateau and lasts 10 min t = time

The measurement period begins 5 min after the onset of plateau and lasts 10 min, as shown in Fig. 2. The plateau was defined as the temperature variation less than 1 °C and relative humidity variation less than 5%. Sample rate: 1 Hz; N: 600 points.

### Statistical analysis

Statistical analysis was performed on absolute humidity.

Mean values for the various situations were compared by analysis of variance using a nonparametric test: Kruskal-Wallis test using Matlab software with statistics toolbox (Mathworks, Natick, MY). A probability level *p* < 0.05 was considered significant.

All configurations were compared to each other one by one for the same humidifier and the various humidifiers were then compared to each other using the same configuration.

## Results

Measurements were performed for a total of 40 configurations at room temperature of 24 °C ± 2 °C and relative humidity of 28% ± 6%.

### Humidifier performances

**Fig. 3:**
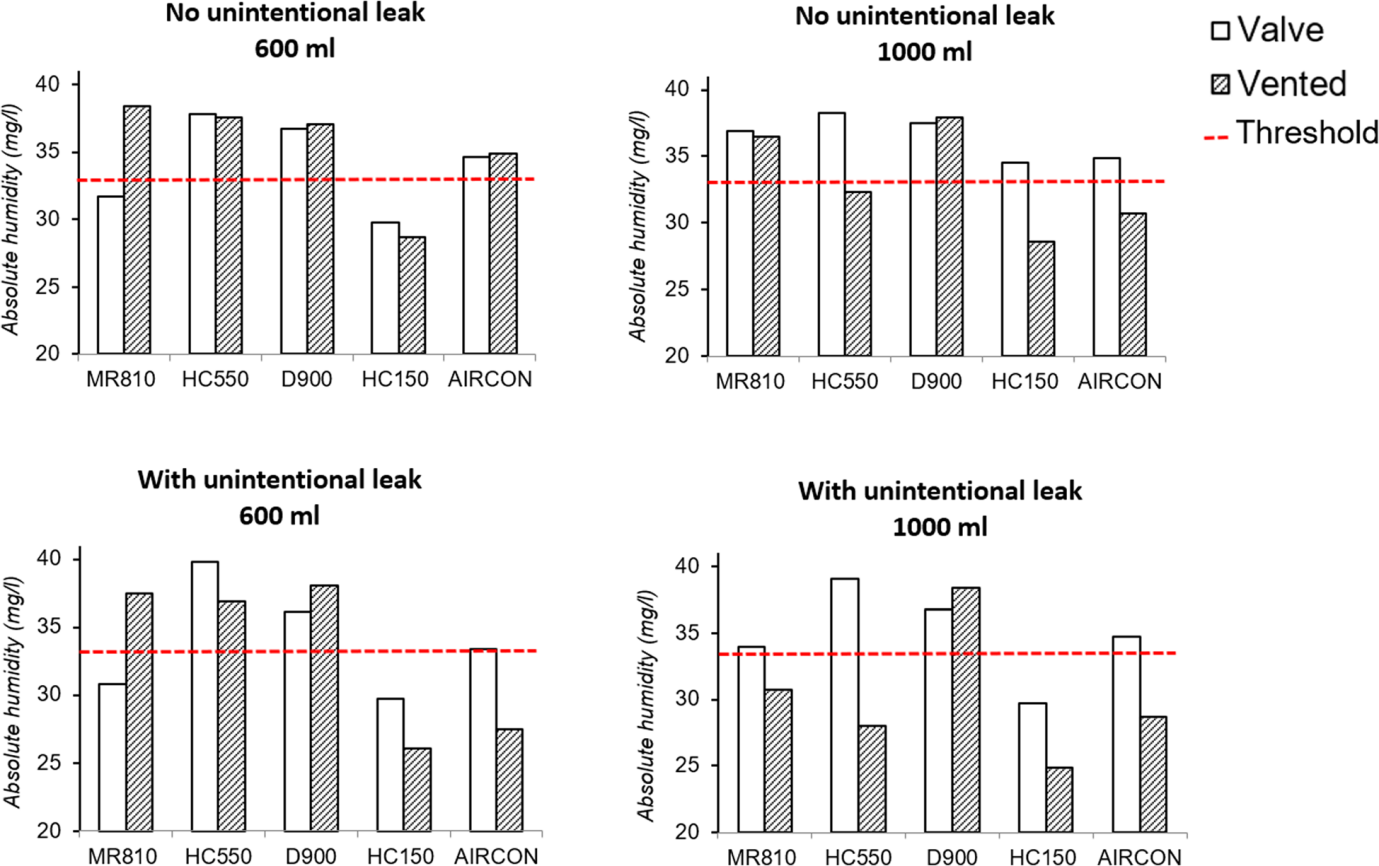
Graph showing absolute humidity in mg/L as a function of the various configurations tested. The absolute humidity (median) is expressed in mg/L for different humidifier (MR810, HC550, D900, HC150 and AIRcon). The valve circuit or vented circuit is watertight = closed (no unintentional leak) or with an unintentional leak. The tidal volume is 600 or 1000 mL. Results were obtained on the set-up comprising 2 hygrometer probes, drying and change of circuits between each configuration except for the AIRcon (set-up with 1 hygrometer probe). All differences were statistically significant with *p* value always lower than 0.001. The threshold corresponds to AARC recommendations (absolute humidity of 33 mg/L). AARC = American Association for Respiratory Care

Absolute humidity results are summarized in Fig. 3 and Additional file 1.

All differences were statistically significant (data not shown).

Absolute humidity (AH) in the valve circuit was between 30 mg/L and 40 mg/L. This humidification was sufficient in all configurations for three humidifiers (HC550, D900 and AIRcon), while the MR810 failed to achieve sufficient humidification in two out of four configurations and the HC150 failed to achieve sufficient humidification in three out of four configurations.

AH below 33 mg/L [19] was observed more frequently with the vented circuit.

For all humidifiers except HC150, under optimal conditions with a tidal volume of 600 mL without unintentional leak, humidification was sufficient (lowest AH was 35 mg/L), but, with a higher tidal volume or an unintentional leak, only the D900 humidifier provided sufficient humidification in all situations (AH equal to 38 mg/L). All other humidifiers failed to achieve sufficient humidification in at least one configuration.

The results of statistical analysis comparing the various situations and the various humidifiers are presented as Additional files 2 and 3.

### Water consumption of the reservoir

**Fig. 4:**
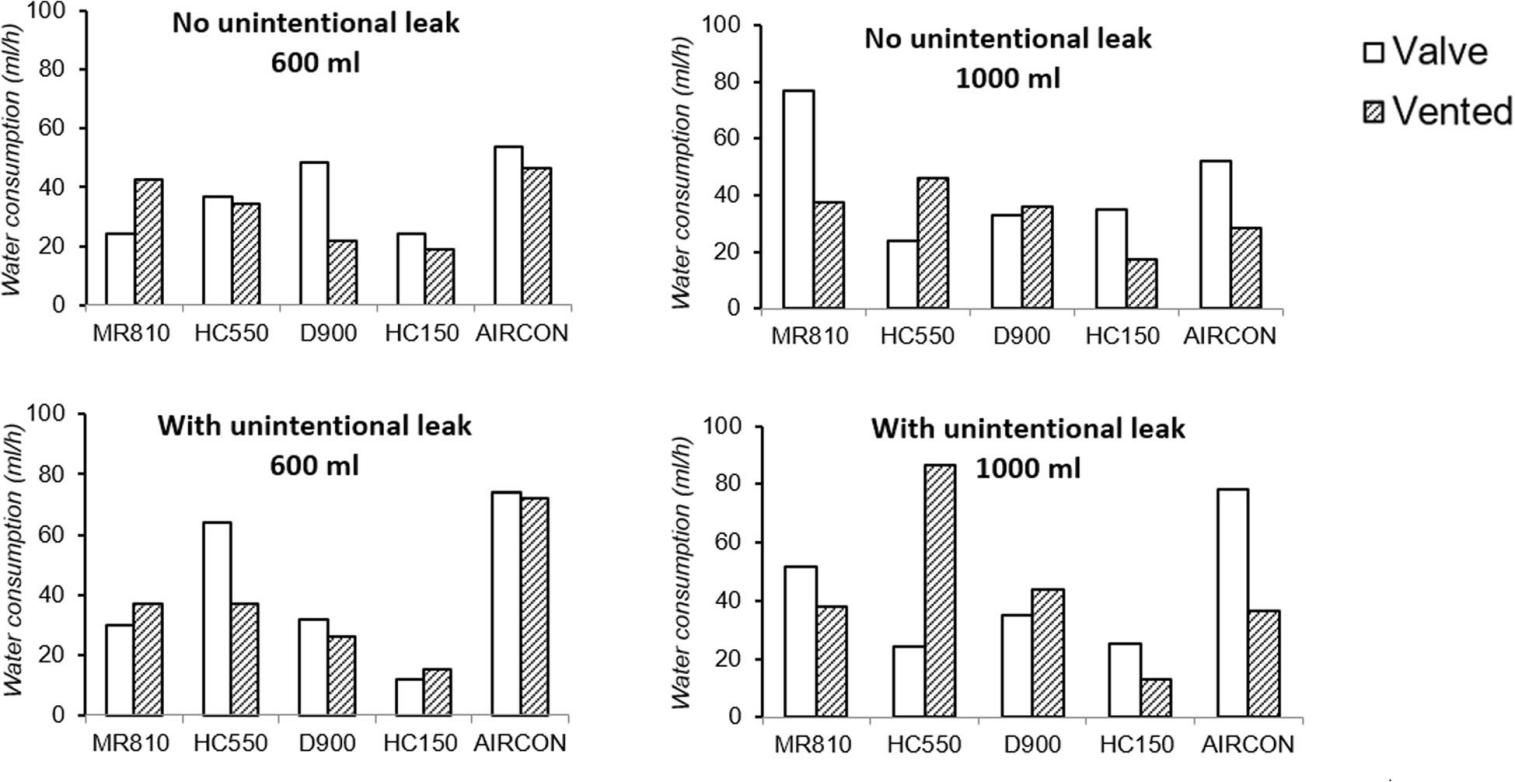
Graph showing water consumption as a function of the various configurations tested. Results of water consumption are expressed in ml/hour for different humidifier (MR810, HC550, D900, HC150 and AIRcon). Results were obtained on the set-up comprising 2 hygrometer probes, drying and change of circuits between each configuration except for the AIRcon (set-up with 1 hygrometer probe). The valve circuit or vented circuit is watertight = closed (with no unintentional leak) or with an unintentional leak. The tidal volume is 600 or 1000 mL

The results are presented in Fig. 4 and Additional files 4 and 5.

In most of the case, water consumption was less than 40 ml/h. For the Aircon, water consumption was the higher (between 28 and 78 ml/h) for all test conditions showing that the water reservoir could be empty in less than 10 h. Water consumption was more variable for the MR810 and HC550, with excessive water consumption in several situations: valve circuit with a high tidal volume for the MR810 (77 ml/h without unintentional leak and 51 ml/h with unintentional leak) and in two conditions for the HC550, especially in the case of an added unintentional leak.

## Discussion

This bench test confirmed our hypothesis that under certain home ventilation conditions it can be difficult to maintain satisfactory humidification. It is due to the limited quality of humidifiers, but also clinical situations inducing very high insufflated flow rates, as in the case of leaks or high tidal volume, for which some of studied humidifiers failed to achieve the recommended humidification objectives. Among all humidifiers included in the study, only one device maintained satisfactory humidification in all simulated conditions, and two devices performed acceptable water consumption.

The main strength of this study is the quality of the test bench. Creation of the test bench was based on research and documentation concerning other existing test benches and required a number of adaptations [4, 21, 25, 28, 29]. To our knowledge, this is the first reported construction of a heated humidifier test bench combining several ventilation configurations, including the possibility of addition of an unintentional leak and the use of various breathing circuits.

A major originality of this study was the use of an environmental chamber around the test lungs to more closely reproduce real-life conditions with a constant temperature of 34 °C corresponding to the temperature of the carina. This is an important point, as the first tests performed without an environmental chamber demonstrated the formation of condensation in the hygrometry probe, which could induce saturation of the probe and inaccurate results. This condensation was due to an excessive temperature difference between the outside and the inside of the circuit. An environmental chamber must be used to ensure more rigorous experimental conditions, although this makes the test bench more complicated, possibly constituting a limiting factor to reproduce this test bench by certain teams or certain manufacturers. In order to further decrease the risks of condensation in the probe, the initial set-up was also modified by adding a second heated measuring probe (high humidity level probe), and the circuits were dried and changed between each configuration. Nevertheless, the test bench is easy to use and is composed of easily accessible equipment.

This study demonstrates, for the first time to our knowledge, that the various home humidifiers currently available on the market may fail to ensure adequate humidification, even if only for a few hours a day, in fairly common conditions such as unintentional leaks or increased tidal volume, despite the clearly defined and strict specifications of the ISO standard [19]. As demonstrated by previous bench tests, humidification performances are decreased in the presence of increased tidal volume or volume-control mode [20–22], as confirmed by the results obtained with home humidifiers. These lower performances can be explained by the fact that higher tidal volume increases the air flow rate in the humidification chamber, resulting in less effective humidification, as a larger volume of air has to be humidified over a given time with a shorter air-water contact time. Air also enters the chamber with a certain degree of turbulence, which has been shown to have a negative impact on humidification [23]. For similar reasons, humidification is also less effective in the presence of an unintentional leak. The HC150 humidifier, indicated for non-invasive ventilation or CPAP (Continuous Positive Airway Pressure) but sometimes used in invasive ventilation, was the only device to present insufficient humidification in almost all conditions. We therefore confirm that a humidifier intended for noninvasive ventilation or CPAP (such as the HC150) cannot provide sufficient humidification during invasive ventilation regardless of the conditions.

An original finding of this study, which can have very important consequences for the patient’s everyday life, was the satisfactory water consumption of the reservoir in most situations, except for one humidifier (AIRcon). This is an important point for home ventilation compared to hospital ventilation, as the patient is less regularly monitored, especially at night, and caregivers are less well trained in the management of tracheostomized patients, especially concerning accessories such as humidifiers. Water consumption has never been previously studied. We decided to express the results in ml/h and time to empty the water reservoir which is an important criterion in home ventilation.

Several limitations of this study need to be addressed. Firstly, it was difficult to maintain a constant room temperature during this study, especially as the presence of the environmental chamber in the room rapidly increased the room temperature. However, several studies have shown that high room temperature can decrease humidifier performance [20, 21, 25, 26], but the temperatures studied in these publications were about 28–30 °C, while the room temperature in our study never exceeded 26 °C with a mean of 24 °C. Secondly, our study used extreme humidifier test conditions, now made possible by major progress in ventilator turbines, but also making humidification more difficult. First of all, unintentional leaks on our test bench were both inspiratory and expiratory and occurred continuously. Under real-life conditions, leaks can be inspiratory and/or expiratory and do not necessarily last the entire duration of expiration or inspiration [30] and may not be continuous throughout the day. Tidal volume was set to 1000 mL, which also corresponds to extreme conditions. However, these extreme conditions can be observed in clinical practice in tracheostomized patients (with leak ventilation for phonation, for example) [31]. Thirdly, the response time of the hygrometers we used is too long to detect inspiratory humidity during respiratory cycle (30 s). What is detected is an average humidity in the respiratory circuit and both inspiratory and expiratory gases influence measurements. However, the purpose of our study was to measure and compare the humidity in the respiratory circuit when it become stable (after a 5-min plateau) and not to separate the inspiratory and expiratory humidity. We believe that the average humidity we measure can be representative of the humidity in the respiratory circuit during long term mechanical ventilation. Fourthly, this study was conducted on a test bench and did not take into account events that may occur during ventilation, such as upper airway obstruction or asynchrony. However, we decided not to add these factors to our simulation bench, as these events can be considered to worsen the situation, which would only have accentuated our results. Fifthly, expiration with test lungs differs from expiration under real-life conditions, in which part of the humidity of expired gases is trapped by airway mucosa [3, 32]. Humidification with test lungs is therefore probably less effective than with true lungs. Sixthly, only five humidifiers were tested in this study, as the objective of this study was not to compare humidifiers in terms of their humidification performances, but to show that humidification may be insufficient in certain extreme situations. We therefore decided to test recent humidifiers from various manufacturers commonly used for invasive ventilation with various characteristics (presence or absence of an autoregulation system). The other humidifiers tested in this study were among the most efficient, but many other types of humidifiers are now available, some of which are integrated into ventilators. Finally, each manipulation was performed only once and measurements only lasted 10 min after reaching a stability of measures (plateau) for 5 min. Each measurement could have been continued for a long time after reaching the plateau, as performed in other bench tests [20, 25], but the stability of the results at the plateau indicates that a longer measuring time would not have changed the results.

Although it is difficult to extrapolate data recorded under in vitro conditions to real-life conditions, despite a test bench that reproduces these conditions as accurately as possible, and knowing that there are no specific recommendations for home invasive ventilation (current recommendations of absolute humidity above 33 mg/L do not differentiate between short-term (intensive care) or long-term (home) invasive ventilation), these results can constitute a basis for further reflection.

At the patient’s bedside, in the presence of atelectasis, repeated infections or tracheostomy tube occlusion, the search for the cause should include the quality of humidification and that the data of this study should be used to identify situations in which humidification may be insufficient. The tools used to measure the humidity produced in the circuit could also be used at the patient’s bedside or for future clinical studies.

We also believe it is justified to warn consumers about the home use of heated humidifiers in tracheostomized patients. Only humidifiers complying with ISO 80601-2-74 [19] should be used, even more for prolonged home ventilation. In extreme conditions (leak ventilation, vented circuit and/or high tidal volume), the heated humidifier should be immediately set to the highest setting and the water level of the reservoir should be monitored more frequently. The choice of humidifier is also essential because, as demonstrated in this study, not all humidifiers ensure sufficient humidification in these situations.

## Conclusion

This study shows that, during home invasive ventilation, heated humidifiers most commonly used can ensure sufficient humidification except under certain extreme, but nevertheless possible, conditions of ventilation.

We tested five humidifiers and several ventilation conditions in this study, but our test bench could also be used to test other humidifiers in many other ventilation conditions and to ensure validation of humidifiers prior to their release onto the market.

## Additional files

Additional file 1: Table of results of absolute humidity (mg/L) in each configuration. Five different heated humidifiers were tested (MR810, HC550, D900, HC150 and AIRcon). Results were obtained on the set-up comprising 2 hygrometer probes, drying and change of circuits between each configuration except for the AIRcon (set-up with 1 hygrometer probe). The valve circuit or vented circuit is watertight = closed (with no unintentional leak) or with an unintentional leak. The tidal volume is 600 or 1000 mL. The p column corresponds to the *p* value of each configuration compared to the reference configuration (valve circuit/closed/600 mL). (DOCX 14 kb)

Additional file 2: Results of the statistical analysis (p value) comparing the various humidifiers. A Kruskal-Wallis test was used to compare mean absolute humidity (mg/L) achieved by the various humidifiers tested (MR810, HC550, D900, HC150 and AIRcon) for each configuration. (DOCX 21 kb)

Additional file 3: Results of statistical analysis (p value) comparing the various configurations. A Kruskal-Wallis test was used to compare mean absolute humidity (mg/L) achieved by the various configurations for each humidifier. (DOCX 24 kb)

Additional file 4: Table of results of water consumption (ml/h) in each configuration. Five different heated humidifiers were tested (MR810, HC550, D900, HC150 and AIRcon). Results were obtained on the set-up comprising 2 hygrometer probes, drying and change of circuits between each configuration except for the AIRcon (set-up with 1 hygrometer probe). The valve circuit or vented circuit is watertight = closed (with no unintentional leak) or with an unintentional leak. The tidal volume is 600 or 1000 mL. (DOCX 14 kb)

Additional file 5: Results of water consumption as time to empty water reservoir (in hours) in each configuration. Five different heated humidifiers were tested (MR810, HC550, D900, HC150 and AIRcon). Results were obtained on the set-up comprising 2 hygrometer probes, drying and change of circuits between each configuration except for the AIRcon (set-up with 1 hygrometer probe). The valve circuit or vented circuit is watertight = closed (with no unintentional leak) or with an unintentional leak. The tidal volume is 600 or 1000 mL. (DOCX 99 kb)

## Abbreviations

AH: absolute humidity
CPAP: continuous positive airway pressure
PEEP: positive expiratory pressure
VAC: volume assist control
