# Supplementary material for: Impact of leaks and ventilation parameters on the efficacy of humidifiers during home ventilation for tracheostomized patients: a bench study
Source: BMC Pulm Med. 2019 Feb 18;19:43. doi: 10.1186/s12890-019-0812-z (PMC6379988; doi:10.1186/s12890-019-0812-z)
Supplement: Supplementary file 1 — Table of results of absolute humidity (mg/L) in each configuration. Five different heated humidifiers were tested (MR810, HC550, D900, HC150 and AIRcon). Results were obtained on the set-up comprising 2 hygrometer probes, drying and change of circuits between each configuration except for the AIRcon (set-up with 1 hygrometer probe). The valve circuit or vented circuit is watertight = closed (with no unintentional leak) or with an unintentional leak. The tidal volume is 600 or 1000 mL. The p column corresponds to the p value of each configuration compared to the reference configuration (valve circuit/closed/600 mL). (DOCX 14 kb) [file 12890_2019_812_MOESM1_ESM.docx]

### Additional file 1: Table of results of absolute humidity (mg/L) in each configuration.

| **Absolute humidity - AH – mg/L** | | | | | | | | |
| --- | --- | --- | --- | --- | --- | --- | --- | --- |
|  | **Valve/closed/600 mL** | | **Valve/closed/1000 mL** | | **Valve/leak/600 mL** | | **Valve/leak/1000 mL** | |
|  | **Median**  **[5-95° percentile]** |  | **Median**  **[5-95° percentile]** | **p** | **Median**  **[5-95° percentile]** | **p** | **Median**  **[5-95° percentile]** | **p** |
| **MR810** | 32 [32-32] | ref. | 37 [37-37] | <0.001 | 31 [31-31] | <0.001 | 34 [33-36] | <0.001 |
| **HC550** | 38 [38-38] | ref. | 38 [38-38] | <0.001 | 40 [39-40] | <0.001 | 39 [39-39] | <0.001 |
| **D900** | 37 [37-37] | ref. | 37 [37-38] | <0.001 | 36 [36-36] | <0.001 | 37 [37-37] | <0.001 |
| **AIRcon** | 35 [34-35] | ref. | 35 [35-35] | <0.001 | 33 [30-33] | <0.001 | 35 [35-35] | <0.001 |
| **HC150** | 30 [30-30] | <0.001 | 35 [34-35] | <0.001 | 30 [30-30] | <0.001 | 30 [30-30] | <0.001 |
|  | **Vented /closed/600 mL** | | **Vented/closed/1000 mL** | | **Vented/leak/600 mL** | | **Vented/leak/1000 mL** | |
|  | **Median**  **[5-95° percentile]** | **p** | **Median**  **[5-95° percentile]** | **p** | **Median**  **[5-95° percentile]** | **p** | **Median**  **[5-95° percentile]** | **p** |
| **MR810** | 38 [38-39] | <0.001 | 36 [35-37] | <0.001 | 37 [37-38] | <0.001 | 31 [31 -31] | <0.001 |
| **HC550** | 38 [38-38] | <0.001 | 32 [32-33] | <0.001 | 37 [37-37] | <0.001 | 28 [28-28] | <0.001 |
| **D900** | 37 [37-37] | <0.001 | 38 [38-38] | <0.001 | 38 [38-38] | <0.001 | 38 [38-38] | <0.001 |
| **AIRcon** | 35 [35-35] | <0.001 | 31 [31-31] | <0.001 | 27 [27-28] | <0.001 | 29 [28-29] | <0.001 |
| **HC150** | 29 [29-29] | <0.001 | 29 [28-29] | <0.001 | 26 [26-26] | <0.001 | 25 [25-25] | <0.001 |

Five different heated humidifiers were tested (MR810, HC550, D900, HC150 and AIRcon).

Results were obtained on the set-up comprising 2 hygrometer probes, drying and change of circuits between each configuration except for the AIRcon (set-up with 1 hygrometer probe).

The valve circuit or vented circuit is watertight = closed (with no unintentional leak) or with an unintentional leak. The tidal volume is 600 or 1000 mL.

The p column corresponds to the p value of each configuration compared to the reference configuration (valve circuit /closed/600 mL).
