# Supplementary material for: Impact of leaks and ventilation parameters on the efficacy of humidifiers during home ventilation for tracheostomized patients: a bench study
Source: BMC Pulm Med. 2019 Feb 18;19:43. doi: 10.1186/s12890-019-0812-z (PMC6379988; doi:10.1186/s12890-019-0812-z)
Supplement: Supplementary file 2 — Results of the statistical analysis (p value) comparing the various humidifiers. A Kruskal-Wallis test was used to compare mean absolute humidity (mg/L) achieved by the various humidifiers tested (MR810, HC550, D900, HC150 and AIRcon) for each configuration. (DOCX 21 kb) [file 12890_2019_812_MOESM2_ESM.docx]

| **Additional file 2**  Results of the statistical analysis (p value) comparing the various humidifiers |  | **HC550** | **D900** | **AIRcon** | **HC150** |
| --- | --- | --- | --- | --- | --- |
| **Valve/closed/600 mL** | **MR810** | <0.001 | <0.001 | <0.001 | <0.001 |
|  | **HC550** |  | <0.001 | <0.001 | <0.001 |
|  | **D900** |  |  | <0.001 | <0.001 |
|  | **AIRcon** |  |  |  | <0.001 |
| **Valve/closed/1000 mL** | **MR810** | <0.001 | <0.001 | <0.001 | <0.001 |
|  | **HC550** |  | <0.001 | <0.001 | <0.001 |
|  | **D900** |  |  | <0.001 | <0.001 |
|  | **AIRcon** |  |  |  | <0.001 |
| **Valve/leak/600 mL** | **MR810** | <0.001 | <0.001 | <0.001 | <0.001 |
|  | **HC550** |  | <0.001 | <0.001 | <0.001 |
|  | **D900** |  |  | <0.001 | <0.001 |
|  | **AIRcon** |  |  |  | <0.001 |
| **Valve/leak/1000 mL** | **MR810** | <0.001 | <0.001 | <0.001 | <0.001 |
|  | **HC550** |  | <0.001 | <0.001 | <0.001 |
|  | **D900** |  |  | <0.001 | <0.001 |
|  | **AIRcon** |  |  |  | <0.001 |
| **Vented /closed/600 mL** | **MR810** | <0.001 | <0.001 | <0.001 | <0.001 |
|  | **HC550** |  | <0.001 | <0.001 | <0.001 |
|  | **D900** |  |  | <0.001 | <0.001 |
|  | **AIRcon** |  |  |  | <0.001 |
| **Vented/closed/1000 mL** | **MR810** | <0.001 | <0.001 | <0.001 | <0.001 |
|  | **HC550** |  | <0.001 | <0.001 | <0.001 |
|  | **D900** |  |  | <0.001 | <0.001 |
|  | **AIRcon** |  |  |  | <0.001 |
| **Vented/leak/600 mL** | **MR810** | <0.001 | <0.001 | <0.001 | <0.001 |
|  | **HC550** |  | <0.001 | <0.001 | <0.001 |
|  | **D900** |  |  | <0.001 | <0.001 |
|  | **AIRcon** |  |  |  | <0.001 |
| **Vented/leak/1000 mL** | **MR810** | <0.001 | <0.001 | <0.001 | <0.001 |
|  | **HC550** |  | <0.001 | <0.001 | <0.001 |
|  | **D900** |  |  | <0.001 | <0.001 |
|  | **AIRcon** |  |  |  | <0.001 |

###

A Kruskal-Wallis test was used to compare mean absolute humidity (mg/L) achieved by the various humidifiers tested (MR810, HC550, D900, AIRcon and HC150) for each configuration.
