# Supplementary material for: Impact of leaks and ventilation parameters on the efficacy of humidifiers during home ventilation for tracheostomized patients: a bench study
Source: BMC Pulm Med. 2019 Feb 18;19:43. doi: 10.1186/s12890-019-0812-z (PMC6379988; doi:10.1186/s12890-019-0812-z)
Supplement: Supplementary file 3 — Results of statistical analysis (p value) comparing the various configurations. A Kruskal-Wallis test was used to compare mean absolute humidity (mg/L) achieved by the various configurations for each humidifier. (DOCX 24 kb) [file 12890_2019_812_MOESM3_ESM.docx]

| **Additional file 3**  Results of statistical analysis (p value) comparing the various configurations | Valve  Closed  1000mL | Valve  Leak  600mL | Valve  Leak  1000mL | Vented  Closed  600mL | Vented  Closed  1000mL | Vented  Leak  600mL | Vented  Leak  1000mL |
| --- | --- | --- | --- | --- | --- | --- | --- |
| MR810 | | | | | | | |
| Valve/closed/600mL | <0.001 | <0.001 | <0.001 | <0.001 | <0.001 | <0.001 | <0.001 |
| Valve/closed/1000mL |  | <0.001 | <0.001 | <0.001 | <0.001 | <0.001 | <0.001 |
| Valve/leak/600mL |  |  | <0.001 | <0.001 | <0.001 | <0.001 | <0.001 |
| Valve/leak/1000mL |  |  |  | <0.001 | <0.001 | <0.001 | <0.001 |
| Vented/closed/600mL |  |  |  |  | <0.001 | <0.001 | <0.001 |
| Vented/closed/1000mL |  |  |  |  |  | <0.001 | <0.001 |
| Vented/leak/600mL |  |  |  |  |  |  | <0.001 |
| HC550 | | | | | | | |
| Valve/closed/600mL | <0.001 | <0.001 | <0.001 | <0.001 | <0.001 | <0.001 | <0.001 |
| Valve/closed/1000mL |  | <0.001 | <0.001 | <0.001 | <0.001 | <0.001 | <0.001 |
| Valve/leak/600mL |  |  | <0.001 | <0.001 | <0.001 | <0.001 | <0.001 |
| Valve/leak/1000mL |  |  |  | <0.001 | <0.001 | <0.001 | <0.001 |
| Vented/closed/600mL |  |  |  |  | <0.001 | <0.001 | <0.001 |
| Vented/closed/1000mL |  |  |  |  |  | <0.001 | <0.001 |
| Vented/leak/600mL |  |  |  |  |  |  | <0.001 |
| D900 | | | | | | | |
| Valve/closed/600mL | <0.001 | <0.001 | <0.001 | <0.001 | <0.001 | <0.001 | <0.001 |
| Valve/closed/1000mL |  | <0.001 | <0.001 | <0.001 | <0.001 | <0.001 | <0.001 |
| Valve/leak/600mL |  |  | <0.001 | <0.001 | <0.001 | <0.001 | <0.001 |
| Valve/leak/1000mL |  |  |  | <0.001 | <0.001 | <0.001 | <0.001 |
| Vented/closed/600mL |  |  |  |  | <0.001 | <0.001 | <0.001 |
| Vented/closed/1000mL |  |  |  |  |  | <0.001 | <0.001 |
| Vented/leak/600mL |  |  |  |  |  |  | <0.001 |
| AIRcon | | | | | | | |
| Valve/closed/600mL | <0.001 | <0.001 | <0.001 | <0.001 | <0.001 | <0.001 | <0.001 |
| Valve/closed/1000mL |  | <0.001 | <0.001 | <0.001 | <0.001 | <0.001 | <0.001 |
| Valve/leak/600mL |  |  | <0.001 | <0.001 | <0.001 | <0.001 | <0.001 |
| Valve/leak/1000mL |  |  |  | <0.001 | <0.001 | <0.001 | <0.001 |
| Vented/closed/600mL |  |  |  |  | <0.001 | <0.001 | <0.001 |
| Vented/closed/1000mL |  |  |  |  |  | <0.001 | <0.001 |
| Vented/leak/600mL |  |  |  |  |  |  | <0.001 |
| HC150 | | | | | | | |
| Valve/closed/600mL | <0.001 | <0.001 | <0.001 | <0.001 | <0.001 | <0.001 | <0.001 |
| Valve/closed/1000mL |  | <0.001 | <0.001 | <0.001 | <0.001 | <0.001 | <0.001 |
| Valve/leak/600mL |  |  | <0.001 | <0.001 | <0.001 | <0.001 | <0.001 |
| Valve/leak/1000mL |  |  |  | <0.001 | <0.001 | <0.001 | <0.001 |
| Vented/closed/600mL |  |  |  |  | <0.001 | <0.001 | <0.001 |
| Vented/closed/1000mL |  |  |  |  |  | <0.001 | <0.001 |
| Vented/leak/600mL |  |  |  |  |  |  | <0.001 |

A Kruskal-Wallis test was used to compare mean absolute humidity (mg/L) achieved by the various configurations for each humidifier.
