# Supplementary material for: Impact of leaks and ventilation parameters on the efficacy of humidifiers during home ventilation for tracheostomized patients: a bench study
Source: BMC Pulm Med. 2019 Feb 18;19:43. doi: 10.1186/s12890-019-0812-z (PMC6379988; doi:10.1186/s12890-019-0812-z)
Supplement: Supplementary file 4 — Table of results of water consumption (ml/h) in each configuration. Five different heated humidifiers were tested (MR810, HC550, D900, HC150 and AIRcon). Results were obtained on the set-up comprising 2 hygrometer probes, drying and change of circuits between each configuration except for the AIRcon (set-up with 1 hygrometer probe). The valve circuit or vented circuit is watertight = closed (with no unintentional leak) or with an unintentional leak. The tidal volume is 600 or 1000 mL. (DOCX 14 kb) [file 12890_2019_812_MOESM4_ESM.docx]

### Additional file 4: Table of results of water consumption (ml/h) in each configuration.

| **Water consumption – ml/h** | | | | |
| --- | --- | --- | --- | --- |
|  | **Valve/closed/600 mL** | **Valve/closed/1000 mL** | **Valve/leak/600 mL** | **Valve/leak/1000 mL** |
| **MR810** | 24 | 77 | 30 | 51 |
| **HC550** | 37 | 24 | 64 | 24 |
| **D900** | 49 | 33 | 32 | 35 |
| **HC150** | 24 | 35 | 12 | 25 |
| **AIRcon** | 54 | 52 | 74 | 78 |
|  | **Vented /closed/600 mL** | **Vented/closed/1000 mL** | **Vented/leak/600 mL** | **Vented/leak/1000 mL** |
| **MR810** | 43 | 37 | 37 | 38 |
| **HC550** | 34 | 46 | 37 | 87 |
| **D900** | 22 | 36 | 26 | 44 |
| **HC150** | 19 | 17 | 15 | 13 |
| **AIRcon** | 47 | 28 | 72 | 37 |

Five different heated humidifiers were tested (MR810, HC550, D900, HC150 and AIRcon).

Results were obtained on the set-up comprising 2 hygrometer probes, drying and change of circuits between each configuration except for the AIRcon (set-up with 1 hygrometer probe).

The valve circuit or vented circuit is watertight = closed (with no unintentional leak) or with an unintentional leak. The tidal volume is 600 or 1000 mL.
