# Supplementary material for: Impact of leaks and ventilation parameters on the efficacy of humidifiers during home ventilation for tracheostomized patients: a bench study
Source: BMC Pulm Med. 2019 Feb 18;19:43. doi: 10.1186/s12890-019-0812-z (PMC6379988; doi:10.1186/s12890-019-0812-z)
Supplement: Supplementary file 5 — Results of water consumption as time to empty water reservoir (in hours) in each configuration. Five different heated humidifiers were tested (MR810, HC550, D900, HC150 and AIRcon). Results were obtained on the set-up comprising 2 hygrometer probes, drying and change of circuits between each configuration except for the AIRcon (set-up with 1 hygrometer probe). The valve circuit or vented circuit is watertight = closed (with no unintentional leak) or with an unintentional leak. The tidal volume is 600 or 1000 mL. (DOCX 99 kb) [file 12890_2019_812_MOESM5_ESM.docx]

### **Additional file 5: Results of water consumption as time to empty water reservoir (h) in each configuration.**

| **Water consumption – Time to empty water reservoir (hour)** | | | | |
| --- | --- | --- | --- | --- |
|  | **Valve/closed/600 mL** | **Valve/closed/1000 mL** | **Valve/leak/600 mL** | **Valve/leak/1000 mL** |
| **MR810** | 17 | 5 | 13 | 8 |
| **HC550** | 11 | 17 | 6 | 17 |
| **D900** | 10 | 15 | 16 | 14 |
| **HC150** | 17 | 11 | 33 | 16 |
| **AIRcon** | 4 | 4 | 3 | 3 |
|  | **Vented /closed/600 mL** | **Vented/closed/1000 mL** | **Vented/leak/600 mL** | **Vented/leak/1000 mL** |
| **MR810** | 9 | 11 | 11 | 11 |
| **HC550** | 12 | 9 | 11 | 5 |
| **D900** | 23 | 14 | 19 | 11 |
| **HC150** | 21 | 24 | 27 | 31 |
| **AIRcon** | 4 | 7 | 3 | 5 |


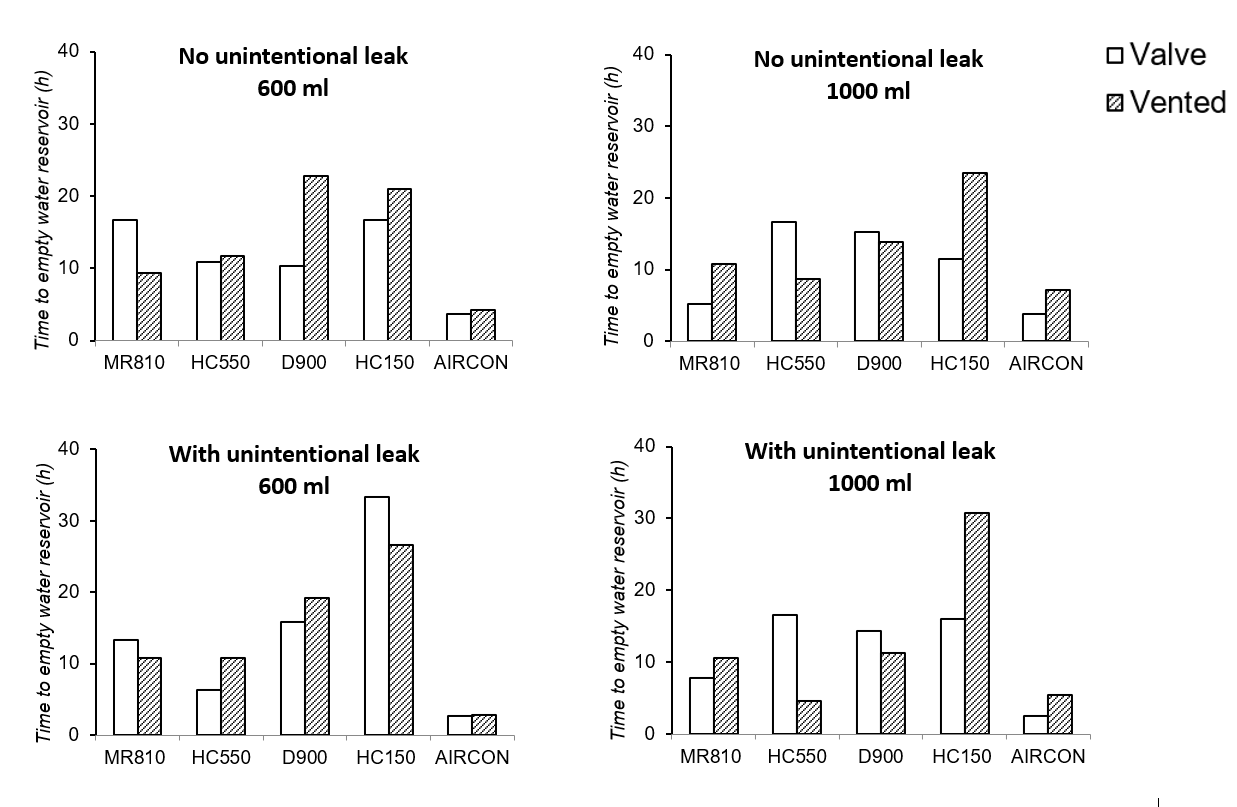


Five different heated humidifiers were tested (MR810, HC550, D900, HC150 and AIRcon).

Results were obtained on the set-up comprising 2 hygrometer probes, drying and change of circuits between each configuration except for the AIRcon (set-up with 1 hygrometer probe).

The valve circuit or vented circuit is watertight = closed (with no unintentional leak) or with an unintentional leak. The tidal volume is 600 or 1000 mL.
